# Dataset on patient education and digital information quality in knee cartilage restoration with matrix-induced autologous chondrocyte implantation (MACI)

**DOI:** 10.1016/j.dib.2025.112353

**Published:** 2025-12-03

**Authors:** Camila Vicioso, Hannah L. Terry, Ava G. Neijna, Sabrina M. Strickland

**Affiliations:** a Department of Orthopaedic Surgery, Icahn School of Medicine at Mount Sinai; b Department of Sports Medicine Orthopaedic Surgery, Hospital for Special Surgery

**Keywords:** Orthopaedic surgery, Online health resources, Patient engagement, Search engine analysis, Digital health literacy, Orthopaedic surgery education, Health communication, Healthcare educational content development

## Abstract

This dataset provides a comprehensive collection and classification of publicly available online questions and linked websites related to matrix-induced autologous chondrocyte implantation (MACI), an implant that can be utilized by orthopaedic surgeons for patients requiring knee cartilage restoration. Eight MACI-related search terms were entered individually into a history-cleared Google Chrome browser in incognito mode to minimize personalization bias. For each term, the “People Also Ask” feature was expanded to retrieve approximately 200 question-website pairs, yielding a total of 1620 entries that were compiled and screened for relevance. The final dataset includes 1107 unique, relevant question–website pairs organized in a spreadsheet containing variables for search term, question text, linked website, website source type, Rothwell classification (Fact, Policy, or Value) and subcategories, JAMA Benchmark Criteria component scores, total JAMA credibility score, and thematic grouping based on question content and author consensus. Each entry was rated independently by two reviewers, with discrepancies resolved by the primary author using an Excel-based verification process. Descriptive statistics and logistic regression were performed in Python (statsmodels, SciPy). The dataset is accompanied by materials outlining classification frameworks, frequently repeated questions, and commonly linked websites. By documenting how patients search for and encounter information on a popular cartilage restoration option, this dataset provides a model for evaluating digital health resources and developing accurate, accessible educational content for patients and clinicians across medical disciplines.

Specifications Table

**Table:** 

Subject	Health Sciences, Medical Sciences & Pharmacology
Specific subject area	Analysis of online information on MACI and development of patient education materials to support clinical decision-making for orthopaedic knee cartilage repair
Type of data	Tables (3), Graph (1), Figure (1), Raw data
Data collection	We entered eight MACI-related search terms into a history-cleared Google browser. The top 200 “People Also Ask” question-website pairs per term were extracted and screened for relevance. Two independent reviewers classified each question by Rothwell category (e.g. technical details, cost, recovery, activities, indications, risks, evaluation, longevity, pain), and each website by source type (academic, medical practice, commercial, government) and JAMA Benchmark Criteria (credibility). The primary author resolved discrepancies. Thematic analysis informed the design of an infographic and patient education website summarizing FAQs and high-credibility sources on MACI*.*
Data source location	Online data was collected. Authors are located at: Icahn School of Medicine at Mount Sinai and the Hospital for Special Surgery. Both institutions are located in New York City, USA.
Data accessibility	**Repository name:** OSF (Project title: Analysis of Online Information on Matrix-Induced Autologous Chondrocyte Implantation (MACI)) **Data identification number:** none **Direct URL to data:**https://osf.io/fnsdx/overview?view_only=dabc90a6fa4e417093d927b752b3a928 **To access the data:** Click on the link above and then download “MACI_GOOGLE_SCRAPE_FOR_ANALYSIS.csv” located under Files Preview.
Related research article	Vicioso C, Neijna A, Terry H, Valdivia L, Wong L, Ren R, Strickland S. **Online Information on MACI Knee Surgery: Analysis and Opportunities to Improve Patient Education and Decision-Making.***The Knee*. November 2025. https://www.sciencedirect.com/science/article/pii/S0968016025002807?via%3Dihub

## 1 Value of the Data

• This article corresponds to our original research article titled Online information on MACI knee surgery: analysis and opportunities to improve patient education and decision-making [1], and provides the complete dataset as well as figures used to inform our manuscript and patient education materials.
• These data provide a structured dataset analyzing public online questions and linked website sources related to Matrix-Induced Autologous Chondrocyte Implantation (MACI), including over 1600 FAQs and associated websites. Question-website pairs were rated for credibility and content type, uniquely capturing how patients seek and access orthopedic information online about cartilage repair, including what they want to know, the questions they have, and the information being promoted to them across the web.
• The dataset applies standardized frameworks—Rothwell Classification [2,3] for question topic ratings and JAMA Benchmark Criteria [3,4] for website credibility—allowing reproducible assessment of online health information credibility and enabling comparison across orthopedic, medical, and surgical procedures.
• This methodology can be replicated to analyze the online landscape of other healthcare topics to inform the development of patient education materials. Researchers can also reuse these data to examine patient information needs, regarding MACI, and other cartilage restoration procedures. This model can also be applied to other medical or surgical topics.
• Clinicians, professional societies, and patient educators can use the categorized question themes and credibility ratings to identify patient knowledge gaps, develop targeted educational materials, guide web-based education strategies, and direct patients toward high-quality, evidence-based online resources about MACI and other cartilage repair options.
• The dataset directly informed the development of a clinician-reviewed infographic and public patient education website (https://orthofaqs.my.canva.site/maci-faqs) summarizing frequently asked questions and credible sources on MACI, providing a reproducible model for translating digital search analytics into practical patient education tools.

## 2 Background

The dataset was compiled to address how patients research cartilage repair options online, particularly Matrix-Induced Autologous Chondrocyte Implantation (MACI)—a relatively newer, cell-based implant for knee cartilage restoration among a growing number of cartilage repair techniques [5]. As a relatively recent advancement, MACI offers unique benefits for select patients, though may remain less familiar to the general public compared to established procedures. This makes accurate and accessible patient education especially important to support informed decision-making and to set realistic expectations about recovery and long-term outcomes. As patients increasingly turn to the internet to guide their choices [[6], [7], [8], [9], [10]], the quality and credibility of available information can strongly influence perceptions of surgical outcomes and candidacy for MACI.

The motivation behind compiling these data was to systematically capture the questions, concerns, and informational gaps that shape patients’ online learning about MACI and to evaluate the credibility of the websites providing those answers. By making this dataset publicly available, the authors aim to support transparency in online health information and to help clinicians, researchers, and industry partners strengthen patient education resources for cartilage restoration procedures.

This dataset also informed the creation of a clinician-reviewed infographic and a publicly accessible patient education website (https://orthofaqs.my.canva.site/maci-faqs) that can also be printed as a handout for clinical use by patients and physicians considering MACI. These resources align with ongoing efforts to promote informed patient decision-making and to improve digital access to accurate, evidence-based information about advanced cartilage repair techniques such as MACI.

This *Data in Brief* article complements the related research manuscript, *Online Information on MACI Knee Surgery: Analysis and Opportunities to Improve Patient Education and Decision-Making*, by providing the complete rated dataset and additional materials from analyses.

## 3 Data Description

The dataset, *MACI_GOOGLE_SCRAPE_FOR_ANALYSIS.csv*, includes over 1000 unique question-website pairs related to MACI, extracted from Google’s “People Also Ask” feature. Each entry contains the following variables: search term, question text, linked website, website source type (Academic, Commercial, Medical Practice, Government), Rothwell classification (Fact: technical details, cost, recovery timeline, and specific activities, Policy: indications/management and risks/complications, or Value: evaluation of surgery, longevity, and pain), and JAMA Benchmark Criteria scores (presence of authorship, attribution, disclosure, date).

The dataset and accompanying materials are publicly available in the Open Science Framework (OSF) repository.

**Fig. 1:**
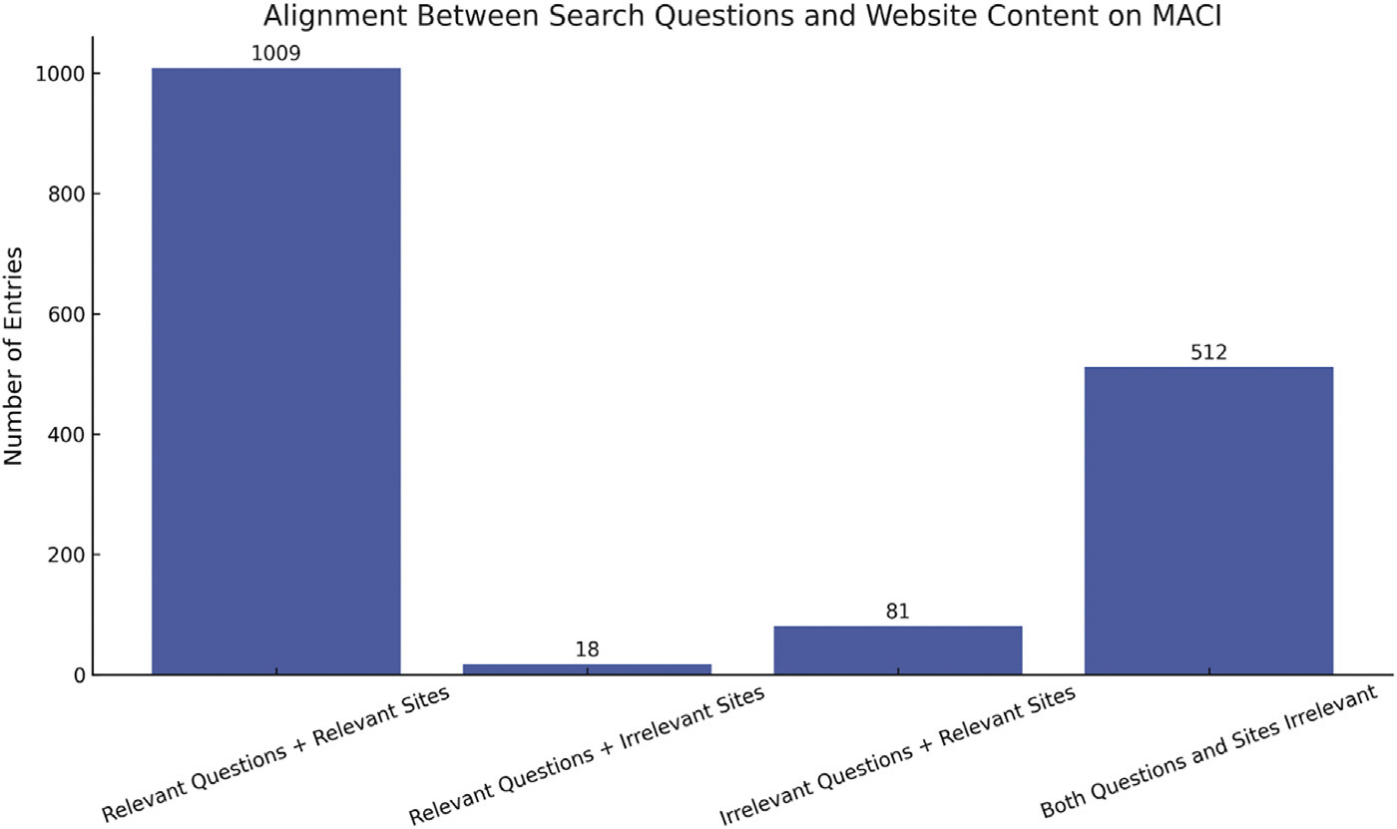
A total of 1620 question-website pairs were extracted from Google’s "People Also Ask" feature. Entries were categorized based on the relevance of both the search question and the linked website to MACI. While most results showed alignment between question and website content, 18 relevant questions led to non-MACI websites, and 81 irrelevant questions linked to MACI-related websites. Among the 512 entries excluded due to both question and website irrelevance, 57.6 % were associated with repeated questions and 71.3 % with repeated websites—indicating that low-relevance content was frequently resurfaced across multiple queries.
• Table 1: Definitions and examples of website source classifications and JAMA Benchmark Criteria used for credibility assessment.

Additional materials to accompany the related research article (*Online Information on MACI Knee Surgery: Analysis and Opportunities to Improve Patient Education and Decision-Making*) include:

• Fig. 1: Diagram of dataset extraction and relevance screening.

**Table 1:** Website source classifications and JAMA Benchmark Criteria used to assess MACI-related search result quality. Website types were categorized as academic, government, medical practice, or commercial. JAMA credibility ratings evaluated authorship, attribution, disclosure, and currency.

	Category	Definition	Examples
**Website Source**	Academic	Content affiliated with academic institutions, academic journals, or university medical centers.	*Northwestern Medicine, Journal of Cartilage & Joint Preservation, Mayo Clinic Sports Medicine, ScienceDirect*
	Medical Practice	Independent physician/practice websites not affiliated with universities.	*Brian Gilmer MD, Ronak Patel MD, Austin Stone MD, All Island Orthopedics, Dr. Burke Orthopedics*
	Commercial	For-profit health info sites, pharma, or commercial content providers.	*Cartilage Repair Blog (Vericel), MACI.com (Vericel), Regenexx*
	Government	Federal or public health websites (e.g., NIH, PubMed, CDC).	*PubMed Central (PMC), National Institutes of Health (NIH), ClinicalTrials.gov, NCBI Bookshelf*
	Social Media	Platforms with user-generated content (e.g., YouTube, Reddit, patient blogs).	— (No example found in this dataset)

**JAMA Benchmark Criteria**	Authorship	Clearly identifies who authored the content.	—
	Attribution	Includes citations, references, or external sources.	—
	Disclosure	Discloses conflicts of interest, funding, or ownership.	—
	Currency (date)	Provides the date of publication or most recent update.	—
• Table 2: Most frequently repeated MACI-related questions (≥20 appearances) with corresponding sources and categories.

**Table 2:** Most frequently repeated MACI-related questions from Google’s “People Also Ask” results. Each question is categorized using Rothwell’s framework, with the most commonly linked website and frequency of appearance shown. These questions appeared ≥20 times. *One non–MACI-specific question is included due to its consistent co-appearance with MACI queries.

Question	Category | Subcategory	Associated Website	No. of Repeats (%)
Who is a good candidate for MACI surgery?	Policy | **Indications/management**	**Cartilage Repair Blog, Vericel Corporation |***Is MACI Knee Cartilage Repair Right for Your Patients?***|**https://www.cartilagerepairblog.com	42 (3.79%)
Is the MACI procedure worth it?	Value **| Evaluation of Surgery**	**Mayo Clinic Sports Medicine |***MACI: Repairing knee cartilage damage***|**https://sportsmedicine.mayoclinic.org	33 (2.98%)
Is MACI surgery arthroscopic?	Fact **| Technical Details**	**Vericel Corporation (MACI) |***Arthroscopy Is the First Step for Durable Knee Cartilage Repair***|**https://www.maci.com	31 (2.8%)
What is the pain after MACI surgery?	Value **| Pain**	**Austin V. Stone, MD |***Post-Op Instructions for Autologous Chondrocyte Implantation (MACI) of Patella/Trochlea***|**https://www.austinstonemd.com	30 (2.71%)
How much does MACI surgery cost?	Fact **| Cost**	**Arthroscopy Journal, Elsevier |***No Clear Winner When Comparing Cost-Effectiveness of Particulated Juvenile Articular Cartilage With Matrix-Induced Autologous Chondrocyte Implantation***|**https://www.arthroscopyjournal.org	30 (2.71%)
What is the success rate of MACI cartilage repair?	Value **| Evaluation of Surgery**	**Journal of Cartilage & Joint Preservation |***New Horizons in Cartilage Repair: Update on Treatment Trends and Outcomes***|**https://www.cartilagejournal.org	29 (2.62%)
How long does a MACI procedure last?	Value **| Longevity**	**Hospital for Special Surgery (HSS) |***Matrix-induced Autologous Chondrocyte Implantation (MACI)***|**https://www.hss.edu	29 (2.62%)
What are the side effects of MACI surgery?	Policy **| Risks/complications**	**Brian Gilmer, MD |***Matrix-induced Autologous Chondrocyte Implantation: MACI***|**https://www.briangilmermd.com	29 (2.62%)
What are the disadvantages of MACI?	Value **| Evaluation of Surgery**	**Vericel Corporation (MACI) |***Time Matters: Why You Shouldn't Wait to Treat Your Cartilage Injury***|**https://www.maci.com	27 (2.44%)
Is MACI procedure FDA approved?	Fact **| Technical Details**	**Investor Relations - Vericel Corporation |***Vericel Announces FDA Approval and Commercial Availability of MACI***|**https://investors.vcel.com	26 (2.35%)
Is MACI procedure covered by insurance?	Fact **| Cost**	**Vericel Corporation (MACI) |***MyCartilageCare, a support program for MACI patients***|**https://www.maci.com	25 (2.26%)
Is MACI a stem cell therapy?	Fact **| Technical Details**	**Dr. Burke Orthopedics |***What Actually Are Stem Cells?***|**https://drburkeortho.com	24 (2.17%)
What is the age limit for MACI procedure?	Policy **| Indications/management**	**NIH / PubMed Central |***New and Emerging Techniques in Cartilage Repair: MACI***|**https://pmc.ncbi.nlm.nih.gov	24 (2.17%)
When can I walk after MACI surgery?	Fact **| Specific activities**	**Vericel Corporation (MACI) |***Sticking to Your Rehab Plan After Knee Cartilage Repair Surgery***|**https://www.maci.com	23 (2.08%)
What is the success rate of MACI?	Value **| Evaluation of Surgery**	**Northwestern Medicine |***Matrix-Induced Autologous Chondrocyte Implantation***|**https://www.nm.org	23 (2.08%)
What is the difference between OATS and MACI procedure?	Fact **| Technical Details**	**Regenexx® at New Regeneration Orthopedics |***Knee Cartilage Healing: Traditional vs. New TreatmentsRegenexx®***|**https://newregenortho.com	22 (1.99%)
What is the recovery time for MACI knee surgery?	Fact **| Recovery timeline**	**Dr. Ronak Patel |***Recovery from Knee Cartilage Restoration Surgery***|**https://www.drronakpatel.com	20 (1.81%)
What is the new treatment for knee cartilage damage?*	**—**	**UC Davis Health |***New implant helps repair knee cartilage in UC Davis Health patients***|**https://health.ucdavis.edu	20 (1.81%)
• Table 3: Most frequently repeated websites with associated FAQs, website types, and frequency of appearance.

**Table 3:** Most frequently repeated websites in Google’s “People Also Ask” results for MACI-related queries. Each entry includes the website source, article title, most commonly associated question, website type, and frequency of appearance. These websites were repeated ≥20 times; non–MACI-related websites were excluded.

Source | Article Title | Link	Associated FAQ	Website Type	No. of Repeats (%)
**Cartilage Repair Blog, Vericel Corporation |***Is MACI Knee Cartilage Repair Right for Your Patients?***|**https://www.cartilagerepairblog.com	Who is a good candidate for MACI surgery?	Commercial	47 (4.24 %)
**Healthline Media |***Knee Cartilage Replacement: 5 Options to Consider***|**https://www.healthline.com	How much does a MACI cartilage repair cost?	Commercial	44 (3.97 %)
**Brian Gilmer, MD |***Matrix-induced Autologous Chondrocyte Implantation: MACI***|**https://www.briangilmermd.com	What are the side effects of MACI surgery?	Medical Practice	42 (3.79 %)
**Northwestern Medicine |***Matrix-Induced Autologous Chondrocyte Implantation***|**https://www.nm.org	What is the success rate of MACI?	Academic	41 (3.7 %)
**Arthroscopy Journal, Elsevier |***No Clear Winner When Comparing Cost-Effectiveness of Particulated Juvenile Articular Cartilage With Matrix-Induced Autologous Chondrocyte Implantation***|**https://www.arthroscopyjournal.org	How much does MACI surgery cost?	Academic	38 (3.43 %)
**Vericel Corporation (MACI) |***Time Matters: Why You Shouldn't Wait to Treat Your Cartilage Injury***|**https://www.maci.com	What are the disadvantages of MACI?	Commercial	35 (3.16 %)
**Vericel Corporation (MACI) |***MyCartilageCare, a support program for MACI patients***|**https://www.maci.com	Is MACI procedure covered by insurance?	Commercial	34 (3.07 %)
**NIH / PubMed Central |***New and Emerging Techniques in Cartilage Repair: MACI***|**https://pmc.ncbi.nlm.nih.gov	What is the age limit for MACI procedure?	Government	34 (3.07 %)
**Hospital for Special Surgery (HSS) |***Matrix-induced Autologous Chondrocyte Implantation (MACI)***|**https://www.hss.edu	How long does a MACI procedure last?	Academic	34 (3.07 %)
**Austin V. Stone, MD |***Post-Op Instructions for Autologous Chondrocyte Implantation (MACI) of Patella/Trochlea***|**https://www.austinstonemd.com	What is the pain after MACI surgery?	Medical Practice	33 (2.98 %)
**Vericel Corporation (MACI) |***Sticking to Your Rehab Plan After Knee Cartilage Repair Surgery***|**https://www.maci.com	When can I walk after MACI surgery?	Commercial	33 (2.98 %)
**Investor Relations - Vericel Corporation |***Vericel Announces FDA Approval and Commercial Availability of MACI***|**https://investors.vcel.com	Is MACI procedure FDA approved?	Commercial	33 (2.98 %)
**Dr. Burke Orthopedics |** A*re You a Candidate for the MACI Procedure?***|**https://drburkeortho.com	Who is a good candidate for MACI?	Medical Practice	33 (2.98 %)
**Journal of Cartilage & Joint Preservation |***New Horizons in Cartilage Repair: Update on Treatment Trends and Outcomes***|**https://www.cartilagejournal.org	What is the success rate of MACI cartilage repair?	Academic	32 (2.89 %)
**Regenexx® at New Regeneration Orthopedics |***Knee Cartilage Healing: Traditional vs. New TreatmentsRegenexx®***|**https://newregenortho.com	What is the difference between OATS and MACI procedure?	Medical Practice	31 (2.8 %)
**Vericel Corporation (MACI) |***Arthroscopy Is the First Step for Durable Knee Cartilage Repair***|**https://www.maci.com	Is MACI surgery arthroscopic?	Commercial	31 (2.8 %)
**Mayo Clinic Sports Medicine |***MACI: Repairing knee cartilage damage***|**https://sportsmedicine.mayoclinic.org	Is the MACI procedure worth it?	Academic	30 (2.71 %)
**All Island Orthopedics |***MACI® Procedure --Cartilage Restoration***|**https://www.allislandortho.com	How long does it take to grow cartilage for MACI?	Medical Practice	29 (2.62 %)
**Vericel Corporation (MACI) |***Undergoing Knee Cartilage Repair? Here’s What You May Expect in the Early Days of Rehab***|**https://www.maci.com	How long does it take to recover from MACI procedure?	Commercial	28 (2.53 %)
**Dr. Ronak Patel |***Recovery from Knee Cartilage Restoration Surgery***|**https://www.drronakpatel.com	What is the recovery time for MACI knee surgery?	Medical Practice	27 (2.44 %)
**Dr. Burke Orthopedics |***What Actually Are Stem Cells?***|**https://drburkeortho.com	Is MACI a stem cell therapy?	Medical Practice	24 (2.17 %)
**Vericel Corporation (MACI) |***MACI Knee Repair and a Return to Running***|**https://www.maci.com	Can you run after MACI surgery?	Commercial	23 (2.08 %)
**UC Davis Health |***New implant helps repair knee cartilage in UC Davis Health patients***|**https://health.ucdavis.edu*	What is the new treatment for knee cartilage damage?	Medical Practice	22 (1.99 %)
**Rothman Orthopaedics |***Knee Cartilage Procedure (MACI/DeNovo/Osteochondral Allograft)***|**https://rothmanortho.com	What is the brace for MACI procedure?	Academic	22 (1.99 %)
**Hospital for Special Surgery (HSS) |***Latest Advances in Cartilage Repair and Regeneration***|**https://www.hss.edu	Does cartilage grow back after surgery?	Academic	21 (1.9 %)

An infographic and publicly available patient education website, developed from these data, summarize frequently asked questions and link to high-credibility online resources. The website can be accessed at this link: https://orthofaqs.my.canva.site/maci-faqs and also printed as a handout for clinical and patient use.

## 4 Experimental Design, Materials and Methods

Eight search terms related to Matrix-Induced Autologous Chondrocyte Implantation (MACI)—“Matrix-Induced Autologous Chondrocyte Implantation,” “MACI Procedure,” “MACI Surgery,” “MACI Knee Cartilage Repair,” “MACI Implant,” “Mini-open MACI,” “Arthrotomy MACI,” and “Arthroscopic MACI”—were selected by the primary and senior authors to represent a range of phrasing styles commonly used by patients when researching MACI online.

Each term was entered individually into a history-cleared Google Chrome browser in incognito mode on the same day to minimize personalization bias. The “People Also Ask” (PAA) drop-down feature on Google was repeatedly expanded by clicking on additional questions to generate approximately 200 unique questions and corresponding websites per search term. The complete list of question-website pairs was then extracted and entered into a spreadsheet.

The dataset was distributed by the primary author to four independent reviewers, with approximately 800 question-website pairs assigned per reviewer to ensure that each pair was independently rated twice by two different individuals. Each question was classified according to the Rothwell framework (Fact, Policy, or Value) and associated subcategories (technical details, cost, recovery timeline, specific activities, indications/management, risks/complications, evaluation of surgery, longevity, and pain), while each linked website was categorized by source type (academic, medical practice, commercial, or government) and evaluated for credibility using the Journal of the American Medical Association (JAMA) Benchmark Criteria (authorship, attribution, disclosure, and currency; total score range 0–4).

After both rounds of independent classification, the primary author compiled all ratings and used an Excel-based formula to identify discrepancies between reviewers. Any differences in classification or scoring were manually reviewed and resolved by the primary author to ensure uniformity. Once all ratings were finalized, the primary author reviewed each category to verify that questions and websites were sorted consistently. The primary author also reviewed all entries to mark duplicates and irrelevant results, resulting in unique question-website pairs for analysis.

Descriptive statistics summarized Rothwell categories, website source types, and JAMA scores. JAMA scores were reported as means. The Kruskal-Wallis test was used to compare JAMA scores across question subcategories and website types, and binary logistic regression was used to identify predictors of high-credibility content (JAMA ≥ 3), with Rothwell subcategory and website source type as independent variables. Statistical significance was defined as *p* < 0.05. Analyses were performed using Python (statsmodels and SciPy libraries).

Following statistical analysis, a thematic grouping was conducted to identify recurring clinical topics of interest, such as surgical approach or technique, cost and insurance, candidacy, postoperative recovery, risks and complications, long-term outcomes, pain, and comparisons to other cartilage restoration procedures. These groupings were developed through author consensus to summarize recurring informational themes and to identify gaps in publicly available patient resources.

A patient-facing infographic and public website were developed to translate these findings into educational tools. Frequently asked questions and representative answers were drawn directly from the dataset and linked to high-credibility websites identified during analysis. The educational website (https://orthofaqs.my.canva.site/maci-faqs) and printable patient handout were designed in Canva and reviewed by the author team to ensure accuracy and clinical relevance.

## Limitations

his study has several limitations. First, we were unable to determine whether a clinician, patient, or other user asked the questions in the Google People Also Ask (PAA) feature. As a result, we cannot fully characterize the perspectives or intentions represented in the dataset. Our analysis reflects only the webpages surfaced by general Google PAA queries and therefore does not capture all MACI-related online content. Future studies may benefit from identifying who is asking each question and examining search patterns across multiple online platforms to obtain more comprehensive data. There was no formal evaluation of the website content to confirm clinical accuracy or completeness. Instead, JAMA Benchmark Criteria Scores were used for credibility. Because JAMA scores are based on authorship, publication date, disclosures, and references, credible sources may score lower. However, the JAMA framework is a widely accepted method for assessing online health information, and the authors reviewed the clinical accuracy of sources recommended from our results. Further, thematic categories were created through author consensus, which may introduce classification bias. Finally, although MACI-related information is broadly accessible online, the product itself is only available in the United States.

## Ethics Statement

The authors have read and follow the ethical requirements for publication in *Data in Brief*, and confirm that the current work does not involve human subjects, animal experiments, or any data collected from social media platforms.

## Credit Author Statement

**Camila Vicioso:** Conceptualization, Methodology, Formal analysis, Investigation, Writing - Original Draft, Writing - Review & Editing, Visualization, Project administration, Funding acquisition. **Hannah Terry:** Investigation, Writing - Original Draft, Writing - Review & Editing, Project administration, Funding acquisition. **Ava Neijna:** Investigation, Writing - Review & Editing, Project administration, Funding acquisition. **Sabrina Strickland:** Supervision, Writing - Review & Editing, Funding acquisition.

## Data Availability

OSF)Analysis of Online Information on Matrix-Induced Autologous Chondrocyte Implantation (MACI) (Original data).
